# Duplication and nonregistration of COVID‐19 systematic reviews: Bibliometric review

**DOI:** 10.1002/hsr2.541

**Published:** 2022-04-21

**Authors:** Jack A. Helliwell, Joe Thompson, Neil Smart, David G. Jayne, Stephen J. Chapman

**Affiliations:** ^1^ Leeds Institute of Medical Research at St. James's University of Leeds Leeds UK; ^2^ Department of General Surgery Royal Devon and Exeter Hospital Exeter UK

**Keywords:** COVID‐19, duplication, registration, systematic review

## Abstract

**Objectives:**

This study examines the conduct of systematic reviews during the early stages of the COVID‐19 pandemic, including compliance to protocol registration and duplication of reviews on similar topics. The methodological and reporting quality were also explored.

**Methods:**

A cross‐sectional, bibliometric study was undertaken of all systematic review manuscripts on a COVID‐19 intervention published between January 1st and June 30th, 2020. Protocol registration on a publicly accessible database was recorded. Duplication was determined by systematically recording the number of reviews published on each topic of analysis. Methodological quality and reporting quality were assessed using the AMSTAR‐2 and PRISMA 2009 instruments, respectively.

**Results:**

Thirty‐one eligible systematic reviews were identified during the inclusion period. The protocol of only four (12.9%) studies was registered on a publicly accessible database. Duplication was frequent, with 15 (48.4%) of the 31 included studies focusing on either hydroxychloroquine (and/or chloroquine) or corticosteroids. Only one study (3.2%) was of “high” methodological quality, four (12.9%) were “low” quality, and the remainder (*n* = 26, 83.9%) were of “critically low” quality. The median completeness of reporting was 20 out of 27 items (74.1%) with a range of 5–26 (interquartile range: 14–23).

**Conclusion:**

Systematic reviews during the early stages of the COVID‐19 pandemic were uncommonly registered, frequently duplicated, and mostly of low methodological quality. In contrast, the reporting quality of manuscripts was generally good but varied substantially across published reports. There is a need for heightened stewardship of systematic review research, particularly during times of medical crisis where the generation of primary evidence may be rapid and unstable.

## 1 INTRODUCTION

Coronavirus disease 2019 (COVID‐19) caused by severe acute respiratory syndrome coronavirus 2 (SARS‐CoV‐2) has affected more than 267 million people worldwide, with more than 5 million deaths recorded by the World Health Organization.^1^ In the early stages of the pandemic, the academic community responded rapidly to this unprecedented situation. By March 24th, 2020, 398 COVID‐19 studies were published in peer‐reviewed journals, most of which were case series aiming to define the disease. These were made rapidly and openly available by editors and publishers.^2^

Since the early stages of the pandemic, an increasing number of interventional studies aiming to inform the treatment and prevention of COVID‐19 have been published. Many of these are reported in high‐impact journals and have informed clinical practice around the world.^3^, ^4^ In parallel, a series of systematic reviews have also emerged. These represent the highest level of evidence capable of changing practice and policy. Their role can be limited, however, in settings where the primary evidence is reactive, rapidly emerging, and unstable. This observation has been alluded to previously, such as during the Zika virus outbreak in 2015.^5^ In situations like this, systematic reviews may be obsolete as early as the point of first publication, leading to a waste of finite research capacity. It is vital that we reflect on whether improvements can be made during the research process at times of medical crisis, particularly in the conduct of systematic review research.

The aim of this study is to examine the conduct of systematic review research during the early stages of the COVID‐19 pandemic. The study specifically aimed to capture publication practices during this initial period of the pandemic, where the generation of new evidence was rapid and uncertainty amongst the academic community was greatest. Specific objectives were to investigate the frequency of preregistration of protocols and the extent to which topics were subsequently duplicated, along with the methodological and reporting quality of subsequent manuscripts.

## 2 METHODS

### 2.1 Ethics and governance

Research ethics committee approval and registration on the PROSPERO database were not required since this manuscript was a review of published literature. The report has been written with reference to the preferred reporting items for systematic reviews and meta‐analyses (PRISMA) 2020 checklist.^6^

### 2.2 Study design

This was a cross‐sectional, bibliometric study of published systematic review manuscripts reporting primary interventional evidence in the context of COVID‐19. For each review, protocol registration on a publicly accessible database was recorded and duplication was examined by assessing the number of reviews identified for each topic of analysis. Methodological quality was assessed using the AMSTAR‐2 checklist.^7^ This is a widely used 16‐item checklist used for methodological appraisal of systematic reviews and has been shown in previous studies to be reliable and valid.^8^ Reporting quality was assessed according to compliance with the PRISMA 2009 checklist,^9^ which is a reporting checklist endorsed by the EQUATOR network and universally accepted by journals responsible for publishing healthcare research. The citation rate of each manuscript was also explored.

### 2.3 Definitions

COVID‐19 describes the disease caused by the SARS‐CoV‐2. A systematic review of primary interventional evidence was defined as a review assessing the benefits or harms of preventative or therapeutic interventions used in healthcare. This may or may not include a statistical meta‐analysis.

### 2.4 Search strategy

Potentially eligible systematic reviews were identified by performing a systematic search of MEDLINE and EMBASE (via OvidSP). This was performed using the search terms shown in Table 1 and was undertaken by a single investigator on June 30th, 2020. Time limits for this search were set between January 1st and June 30th, 2020. The results were saved offline, and any duplicates were removed. Two independent investigators screened titles, abstracts, and full texts for possible inclusion (Jack A. Helliwell and Joe Thompson), with discrepancies addressed through reexamination and discussion with a third independent investigator (Stephen J. Chapman). Reference lists of included studies were reviewed.

**Table 1:** COVID‐19 interventional systematic review search strategy (June 30th, 2020)

	Search term
1	SARS‐CoV‐2
2	nCoV‐19
3	2019‐nCoV
4	COVID‐19
5	Novel coronavirus
6	Severe acute respiratory syndrome coronavirus 2
7	1 OR 2 OR 3 OR 4 OR 5 OR 6
8	Systematic
9	Review
10	8 OR 9
11	7 AND 10
12	[Remove duplicates]
13	[Jan 2020–current (June 30th, 2020)]
14	[Limit to English]

### 2.5 Eligibility criteria

To be eligible for inclusion, studies were required to be a systematic review or a scoping review with a systematic search and have subject material focusing on an intervention (prevention or treatment) in the context of COVID‐19. Original research articles presenting primary data were excluded, as were editorials and gray literature (conference extracts and other nonpeer‐reviewed work). Articles published in non‐English languages were also excluded since resources to translate these were not available.

### 2.6 Study outcomes

Registration and duplication of review topics were explored. Registration at any time was assessed by examining manuscripts for a unique registry identifier. Where no identifier was found, the PROSPERO database was manually searched. This is a widely used, international database of registered systematic reviews with a health‐related outcome. It was assumed that the absence of an identifier and no record on the PROSPERO database indicated the absence of registration. To assess duplication of research topics, a qualitative consensus process was undertaken in which the scope and final study inclusion of each review were examined and thematically defined by two independent investigators (Jack A. Helliwell and Joe Thompson), with referral to a third investigator for disagreements (Stephen J. Chapman). Other outcomes of interest were methodological and reporting quality of manuscripts. Methodological quality was assessed using the AMSTAR‐2 checklist, which provides an assessment of “critical” and “non‐critical” weaknesses along with an overall measure of “confidence” (Table 2).^7^ Completeness of reporting was assessed using the 27‐item PRISMA 2009 checklist.^9^ The presence of each item was recorded and reviews were assigned a score out of 27. Items that were not applicable (i.e., relating to meta‐analysis only) were assigned a positive score by default to preserve comparability. Finally, the citation history of each manuscript was collected through Google Scholar (https://scholar.google.com) to explore the impact of each review.

**Table 2:** AMSTAR‐2 items and overall confidence

AMSTAR‐2		AMSTAR‐2 overall confidence	
Item 1	Research question and inclusion criteria include PICO	High	No or one noncritical weakness
Item 2^a^	Protocol was registered before the commencement of the review	Moderate	More than one noncritical weakness
Item 3	Explanation of study designs for inclusion	Low	One critical flaw with or without noncritical weaknesses
Item 4^a^	Adequacy of literature search	Critically low	More than one critical flaw with or without noncritical weaknesses
Item 5	Study selection performed in duplicate		
Item 6	Data extraction performed in duplicate		
Item 7^a^	Justification for excluding individual studies		
Item 8	Detailed description of Included studies		
Item 9^a^	Risk of bias from individual studies being included in the review		
Item 10	Source of funding of included studies		
Item 11^a^	Appropriateness of the meta‐analytical methods		
Item 12	Consideration of risk of bias on evidence synthesis		
Item 13^a^	Consideration of risk of bias when interpreting the results of the review		
Item 14	Explanation for and description of any heterogeneity observed		
Item 15^a^	Assessment of the presence and likely impact of publication bias		

Abbreviation: PICO, patient/population, intervention, comparison, and outcomes.

^a^ Critical domain.

### 2.7 Data extraction

Data were extracted from included manuscripts by two independent investigators (Jack A. Helliwell and Joe Thompson) and checked by a third independent investigator (Stephen J. Chapman) in cases of disagreement. Data points of interest were a country of origin (according to the corresponding institution), journal of publication, journal impact factor (2019) according to Thomas Reuters Journal Citation Reports, study population (adult patients, healthcare workers), study domain (prevention, treatment), inclusion period (period within which manuscripts were included), protocol registration on a publicly accessible database, and date of publication.

### 2.8 Statistical analysis

Data are presented descriptively. Registration of reviews at any time and the number of reviews within each topic area are described as a percentage of the total number of studies included. Measures of methodological quality are described both as a proportion of studies within each methodological confidence category and according to adherence to AMSTAR‐2 critical domains. Reporting data using the PRISMA 2009 checklist was summarized to provide an overall score out of 27 for each manuscript. Reporting quality was then described using median and the interquartile range (IQR) across included studies. Standardized citation rates per month were calculated by dividing the total number of citations by the number of months since the first publication. All statistical analysis was undertaken in Microsoft Excel V16.55.

## 3 RESULTS

### 3.1 Demographics

**Figure 1:**
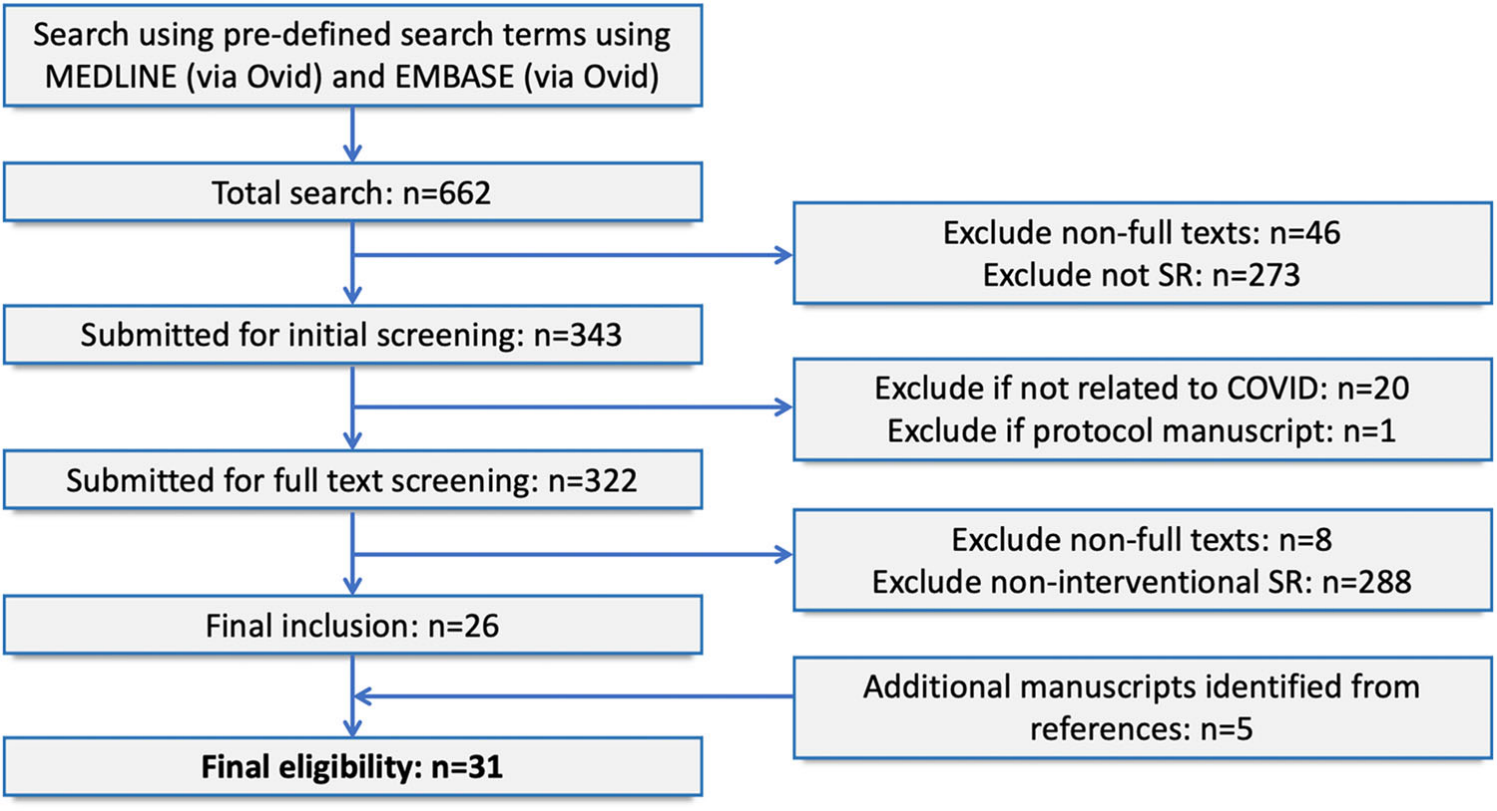
Preferred reporting items for systematic reviews (SRs) and meta‐analyses flow diagram. A protocol manuscript refers to a publication describing a planned or ongoing study

A total of 662 studies were identified from the initial search. Of these, 26 were eligible for inclusion, with another 5 identified from a review of references. A total of 31 studies were eligible for analysis (Figure 1). A list of included studies is available in Table S1. The majority of reviews were performed in the United Kingdom (*n* = 9, 29.0%) and the United States (*n* = 8, 25.8%). Twelve (38.7%) included a statistical meta‐analysis. All but one study focused on a therapeutic treatment (*n* = 30, 96.8%) with the other remaining study focusing on prevention. The most common interventions were hydroxychloroquine and/or chloroquine (*n* = 10, 32.3%), corticosteroids (*n* = 5, 16.1%), and antiviral therapies (*n* = 3, 9.7%) (Table 3).

**Table 3:** Bibliometric characteristics of included studies

Country of publication^a^	COVID‐19 systematic review (SR) (*n* = 31)
United Kingdom	9 (29.0%)
United States	8 (25.8%)
Netherlands	3 (9.7%)
Switzerland	2 (6.5%)
Canada	2 (6.5%)
China	2 (6.5%)
India	2 (6.5%)
Australia	1 (3.2%)
New Zealand	1 (3.2%)
Iran	1 (3.2%)
Study design	
SR	19 (61.3%)
SR + meta‐analysis	12 (38.7%)
Type of intervention	
Treatment	30 (96.8%)
Prevention	1 (3.2%)
Study focus	
Hydroxychloroquine and/or chloroquine	10 (32.3%)
Corticosteroids	5 (16.1%)
Antiviral therapies	3 (9.7%)
Angiotensin‐converting enzyme inhibitors	2 (6.5%)
Tocilizumab	2 (6.5%)
Convalescent plasma	2 (6.5%)
Chinese or herbal medicine	2 (6.5%)
Immunotherapy	1 (3.2%)
Nonsteroidal anti‐inflammatory drugs (NSAIDs)	1 (3.2%)
Face coverings	1 (3.2%)
High flow nasal oxygen	1 (3.2%)
NSAIDs and corticosteroids	1 (3.2%)
Population	
Adult patients	30 (96.8%)
Healthcare workers	1 (3.2%)
Citation rate (per month)	
0–5	7 (22.6%
5.1–10	13 (41.9%)
10.1–15	5 (16.1%)
15.1–20	2 (6.5%)
20.1–25	2 (6.5%)
>25	2 (6.5%)

^a^ Determined according to the corresponding institution

### 3.2 Registration

Protocol registration on a publicly accessible database was disclosed in 4 out of 31 studies (12.9%). The databases used included: PROSPERO (*n* = 2), Centre of Open Science (*n* = 1), and ResearchRegistry (*n* = 1). A further three studies included information about a review protocol within the methods section of the manuscript but did not provide detail as to whether this was available publicly before the completion of the study. Two studies reported that a protocol had not been registered due to the urgency of the public health crisis.

### 3.3 Duplication

**Figure 2:**
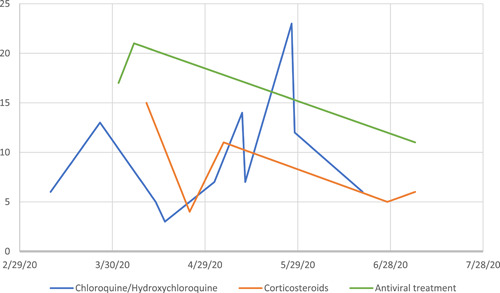
Number of included primary studies within systematic reviews according to date of publication. Interventions with ≥3 published systematic reviews within the study period are shown in the graph

Fifteen (48.4%) out of the 31 included studies focused on either hydroxychloroquine (and/or chloroquine) or corticosteroids as treatment options for COVID‐19 (Table 3). There was a total of 10 reviews of hydroxychloroquine (and/or chloroquine) published between January 1st and June 30th, 2020. Each review had similar time limits for the inclusion of primary studies, with upper bound limits ranging from March to June 2020). Within the reviews of hydroxychloroquine (and/or chloroquine), the median number of included primary studies was 7 (IQR: 6–13, with an overall range of 3–23. There were five reviews of corticosteroids published between April and June 2020. Review search strategies had similar upper bound time limits for inclusion, ranging from March to June 2020. The median number of included primary studies within reviews of corticosteroids was 6 (IQR: 5–11), with an overall range of 4–15. Further inspection of the number of included studies within each review did not show a general increase in the number of included studies over time (Figure 2).

### 3.4 Methodological quality

**Figure 3:**
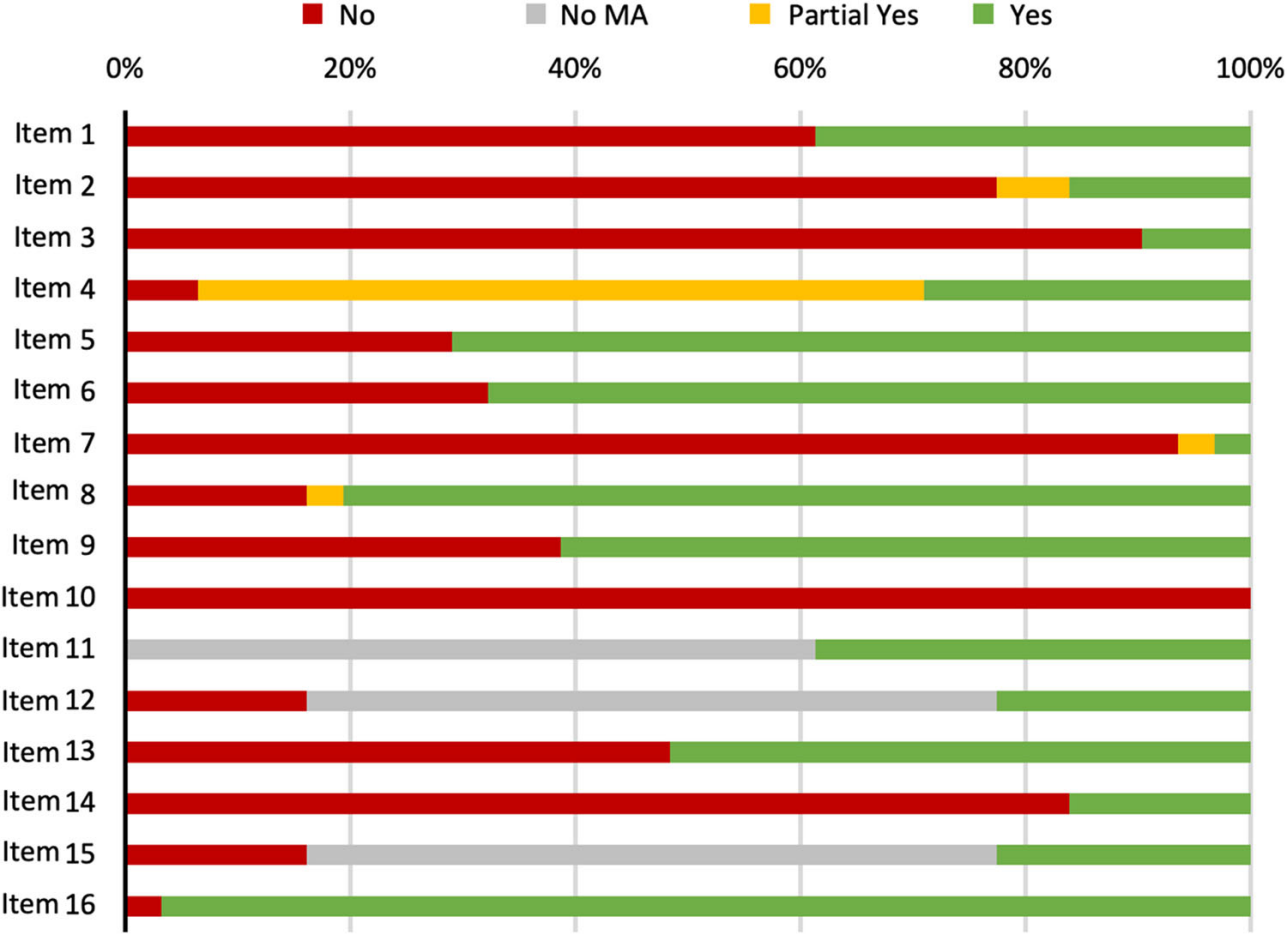
Adherence to each item in the AMSTAR‐2 checklist

Of 31 systematic reviews, only one (3.2%) was of “high” methodological quality, four (12.9%) were “low” quality and the remainder (*n* = 26, 83.9%) were of “critically low” quality. Considering each of the seven critical domains (Figure 3), seven (22.6%) systematic reviews included a review protocol (Item 2) and two (6.5%) included justification for the exclusion of individual studies (Item 7). Nineteen (61.3%) reviews considered risk of bias within individual studies (Item 9) and 16 (51.6%) considered risk of bias when interpreting the results of the review (Item 13). There were 29 (93.5%) which used a comprehensive literature search (Item 4). Of 12 meta‐analyses included, all used appropriate statistical methods (Item 11), and 7 (58.3%) were assessed for the presence and impact of publication bias (Item 15).

### 3.5 Completeness of reporting

The median completeness of reporting across 31 included studies was 20 out of 27 items (74.1%) with a range of 5–26 (IQR: 14–23). Compliance with individual items was variable (Table 4). All studies included background and rationale (Item 3; 100%) and a general interpretation of results (Item 26; 100%). Compliance was greater than 90% for identification of the report as a systematic review within the title (Item 1; 93.5%), description of all sources and date last searched (Item 7; 96.8%), presentation of characteristics of each study (Item 18; 93.5%), and a summary of main findings (Item 24; 90.3%). Compliance was lowest for items: description of objectives (Item 4; 12.9%); statement of review protocol and registration (Item 5; 29%); and results from additional analyses (Item 23, 29%).

**Table 4:** Compliance with PRISMA checklist items

Item	Section/topic	Compliance (*n* = 31)
1	Title	29 (93.5%)
2	Structured summary	20 (64.5%)
3	Rationale	31 (100%)
4	Objectives	4 (12.9%)
5	Protocol and registration	9 (29%)
6	Eligibility criteria	23 (74.2%)
7	Information sources	30 (96.8%)
8	Search	26 (83.9%)
9	Study selection	26 (83.9%)
10	Data collection process	23 (74.2%)
11	Data items	17 (54.8%)
12	Risk of bias in individual studies	19 (61.3%)
13	Summary measures	15 (48.4%)
14	Synthesis of results	18 (58.1%)^a^
15	Risk of bias across studies	15 (48.4%)
16	Additional analyses	10 (32.3%)^a^
17	Study selection	25 (80.6%)
18	Study characteristics	29 (93.5%)
19	Risk of bias within studies	17 (54.8%)
20	Results of individual studies	26 (83.9%)
21	Synthesis of results	11 (91.7%)^a^
22	Risk of bias across studies	15 (48.4%)
23	Additional analyses	9 (29.0%)^a^
24	Summary of evidence	28 (90.3%)
25	Limitations	23 (74.2%)
26	Conclusions	31 (100%)
27	Funding	21 (67.7%)

Abbreviation: PRISMA, preferred reporting items for systematic reviews and meta‐analyses.

^a^ Item only applicable to meta‐analyses.

### 3.6 Citations

Of the 31 included studies, the median total number of citations was 109, with a range of 23–974 (IQR: 67–161). The median citation rate per month was 8, with a range of 2–70 (IQR: 6–13) (Table 3).

## 4 DISCUSSION

### 4.1 Statement of principle findings

A large number of systematic review manuscripts were published in a short period of time during the early stages of the pandemic, many of which have been highly cited by subsequent research outputs. Registration of review protocols on a publicly accessible database was uncommon and duplication of reviews was frequent, with 15 of the included manuscripts focusing on two topics of analysis (hydroxychloroquine/chloroquine and corticosteroids). The majority of reviews showed evidence of notable methodological weaknesses, particularly relating to registration and consideration of study eligibility criteria. In contrast, the reporting of systematic review manuscripts according to guidelines set by the PRISMA 2009 statement was generally good, although this varied substantially across published reports.

### 4.2 Meaning of the study (possible explanations and implications for clinicians and policymakers)

The research community responded rapidly to the COVID‐19 pandemic in 2020 by generating timely and transparent research data. In the early stages, this mostly focused on clinical descriptions of the disease and epidemiological trends. Editorial times were shorter and publishers widened the provision of open access publication.^2^ In contrast, challenges in the conduct and reporting of research also emerged, such as misrepresentation of preprint materials and inadvertent/unnecessary duplication of studies.^10^ The present study highlights other important challenges in the justification, quality, and reporting of systematic reviews produced during the COVID‐19 pandemic. There were as many as 10 published reviews on a single topic of analysis (hydroxychloroquine/chloroquine) within a short 4‐month period. This may be due to very low levels of review protocol registration on publicly accessible databases, which is a problem also encountered in non‐COVID literature.^11^ Strikingly, all but one published systematic review was of low or critically low methodological quality. Similar observations have been made in non‐COVID literature, such as in Pain Medicine, where a previous study demonstrated a median AMSTAR score of 6 out of 11 (IQR: 4–7) and median compliance with PRISMA 2009 of 15.5 out of 27 (IQR: 15–22).^12^ The present findings are important for authors and editorial teams since they depict a unique case scenario where the challenges of nonregistration and review quality may be immediately detrimental to the diffusion of knowledge in clinical practice. It should encourage a heightened shared role for editors and authors in the critical appraisal of systematic review submissions during times of public health crisis, where submissions may increase substantially. One possible solution may be an evidence‐based approach to peer review, such as the assessment of manuscripts using the AMSTAR‐2 critical appraisal tool.^7^ While authors must be applauded for their energy in generating research data rapidly, care must be taken to ensure secondary research reports (i.e., systematic reviews) are time‐justified, robustly conducted, and well‐reported. This is important for ensuring other primary research is not undermined and to maintaining public trust in new and rapidly emerging science. Improved understanding of open‐access registration platforms (such as PROSPERO) may enable improved communication between investigators and promote greater collaboration.^13^ Mandatory registration on such platforms (as is the case for clinical trials) may be one means of increasing registration uptake.

### 4.3 Strengths and weaknesses of the study

Strengths of this study are recognized. The results provide a unique perspective on an existing problem that is evidently exaggerated during times of public health crisis. The results should guide authors and editorial teams during future crises where similar trends in academic publishing may emerge. Weaknesses of the study are also recognized. The study inclusion period was intentionally short to capture a snapshot of time during the early stages of the pandemic. This is a time where uncertainty amongst authors and editorial teams is greatest and where trends and behaviors are possibly most modifiable. The necessary adaptations and learning curves associated with these challenges are important and should be explored through shared experiences between authors and editorial teams. Future studies should consider similar assessments of systematic review research to assess change over time. Second, during the conduct of this study, new guidelines for reporting systematic reviews (PRISMA 2020) were published.^6^ While this is in contrast to the present study, the principles and key take‐home messages generated by this study are considered to be transferable. Lastly, it is notable that only English language reviews were included in this study due to the unavailability of translation sources. It is possible that the results underrepresent the extent of the problem since reviews in other non‐English are likely to exist but were not included in the present analysis.

## 5 CONCLUSION

In conclusion, this review identifies a modifiable problem relating to the conduct of systematic review research during a public health crisis and demonstrates the need for heightened stewardship to ensure only the best evidence informs clinical practice. It highlights particular issues relating to low levels of review protocol registration and high levels of duplication in published reports of systematic reviews. These results add further support for the registration of systematic reviews using open‐access platforms such as PROSPERO. The results should also guide editorial teams during their review and decision‐making processes in times of public health crises. Particular attention should be given to the justification for systematic reviews, particularly in settings where the primary evidence is likely to be highly unstable and rapidly superseded.

## CONFLICTS OF INTEREST

The authors declare no conflicts of interest.

## AUTHOR CONTRIBUTIONS

Stephen J. Chapman and Neil Smart conceptualized the study. Jack A. Helliwell and Joe Thompson performed searches and extracted data, which were verified by Stephen J. Chapman. Jack A. Helliwell prepared the initial manuscript draft, which was subsequently edited by all authors. All authors agreed to the submission. Stephen J. Chapman is the study guarantor.

## Supporting information

Supporting information.

## Data Availability

Data are available upon reasonable request.

## References

[1] WHO . Coronavirus disease 2019 (COVID‐19) situation reports. 2021. Accessed February 14, 2021. https//www.who.int/emergencies/diseases/novel-coronavirus-2019/situation-reports

[2] Helliwell JA , Bolton WS , Burke JR , Tiernan JP , Jayne DG , Chapman SJ . Global academic response to COVID‐19: cross‐sectional study. Learn Publ. 2020; 33(4):385–393. 10.1002/leap.1317 PMC7362145 32836910

[3] Voysey M , Clemens SAC , Madhi SA , et al. Safety and efficacy of the ChAdOx1 nCoV‐19 vaccine (AZD1222) against SARS‐CoV‐2: an interim analysis of four randomised controlled trials in Brazil, South Africa, and the UK. Lancet. 2021;397:99‐111. 10.1016/S0140-6736(20)32661-1 33306989 PMC7723445

[4] RECOVERY Collaborative G , Horby P , Lim WS , et al. Dexamethasone in hospitalized patients with Covid‐19—preliminary report. N Engl J Med. 2020;384:693‐704. 10.1056/nejmoa2021436 32678530 PMC7383595

[5] Brock J . Out of date before it's published. Nat Index. 2019.

[6] Page M , McKenzie J , Bossuyt P , et al. The PRISMA 2020 statement: an updated guideline for reporting systematic reviews. BMJ. 2021;372:n71. 10.1136/bmj.n71 33782057 PMC8005924

[7] Shea BJ , Reeves BC , Wells G , et al. AMSTAR 2: a critical appraisal tool for systematic reviews that include randomised or non‐randomised studies of healthcare interventions, or both. BMJ. 2017;358:j4008. 10.1136/bmj.j4008 28935701 PMC5833365

[8] Lorenz RC , Matthias K , Pieper D , et al. A psychometric study found AMSTAR 2 to be a valid and moderately reliable appraisal tool. J Clin Epidemiol. 2019;114:133‐140. 10.1016/j.jclinepi.2019.05.028 31152864

[9] Liberati A , Altman DG , Tetzlaff J , et al. The PRISMA statement for reporting systematic reviews and meta‐analyses of studies that evaluate healthcare interventions: explanation and elaboration. BMJ. 2009;339:b2700. 10.1136/bmj.b2700 19622552 PMC2714672

[10] Glasziou PP , Sanders S , Hoffmann T . Waste in covid‐19 research. BMJ. 2020;369:m1847. 10.1136/bmj.m1847 32398241

[11] Nepogodiev D , Chapman SJ , Smart NJ , Pinkney TD . Meta‐analysis protocols should be prospectively registered. Tech Coloproctol. 2017;21(6):483‐485. 10.1007/s10151-017-1602-3 28374063

[12] Riado Minguez D , Kowalski M , Vallve Odena M , et al. Methodological and reporting quality of systematic reviews published in the highest ranking journals in the field of pain. Anesth Analg. 2017;125(4):1348‐1354. 10.1213/ANE.0000000000002227 28678074

[13] Booth A , Clarke M , Dooley G , et al. The nuts and bolts of PROSPERO: an international prospective register of systematic reviews. Syst Rev. 2012;1(1):2. 10.1186/2046-4053-1-2 22587842 PMC3348673

