# Supplementary material for: Duplication and nonregistration of COVID‐19 systematic reviews: Bibliometric review
Source: Health Sci Rep. 2022 Apr 21;5(3):e541. doi: 10.1002/hsr2.541 (PMC9059200; doi:10.1002/hsr2.541)
Supplement: Supplementary file 1 — Supporting information. [file HSR2-5-e541-s001.docx]

| **ID** | **Study Title** | **Journal** | **Date of publication** | **Citation rate** |
| --- | --- | --- | --- | --- |
| 1 | Use of corticosteroids in Coronavirus disease 2019 pneumonia: A systematic review of the literature. | Frontiers in Medicine | 24/04/2020 | 9.2 |
| 2 | ACEI/ARB use and risk of infection or severity or mortality of COVID-19: A systematic review and meta-analysis. | Pharmacological research | 15/05/2020 | 9.4 |
| 3 | Supportive Treatment with Tocilizumab for COVID-19: A Systematic Review. | Journal of Clinical Virology | 21/04/2020 | 8.7 |
| 4 | The possible of immunotherapy for COVID-19: A systematic review. | International Immunopharmacology | 02/04/2020 | 6.9 |
| 5 | Chloroquine and hydroxychloroquine in the treatment of COVID-19 with or without diabetes: A systematic search and a narrative review with a special reference to India and other developing countries. | Diabetes and Metabolic Syndrome | 26/03/2020 | 27.6 |
| 6 | A systematic review on the efficacy and safety of chloroquine for the treatment of COVID-19. | Journal of Critical Care | 10/03/2020 | 69.6 |
| 7 | A Rapid Systematic Review of Clinical Trials Utilizing Chloroquine and Hydroxychloroquine as a Treatment for COVID-19. | Society of Academic Emergency Medicine | 02/05/2020 | 8.0 |
| 8 | Systematic review of the efficacy and safety of antiretroviral drugs against SARS, MERS or COVID-19: initial assessment. | Journal of Internal Aids Society | 01/04/2020 | 8.1 |
| 9 | Virological and Clinical Cure in Covid-19 Patients Treated with Hydroxychloroquine: A Systematic Review and Meta-Analysis. | Journal of Medical Virology | 16/04/2020 | 12.4 |
| 10 | QT prolongation, torsades de pointes and sudden death with short courses of chloroquine or hydroxychloroquine as used in COVID-19: a systematic review. | Heart Rhythm | 11/05/2020 | 7.8 |
| 11 | Efficacy and Safety of Integrated Traditional Chinese and Western Medicine for Corona Virus Disease 2019 (COVID-19): a systematic review and meta-analysis. | Pharmacological Research | 11/05/2020 | 4.5 |
| 12 | "Hydroxychloroquine in patients with COVID-19: A Systematic Review and meta-analysis.". | Diabetes and Metabolic Syndrome | 12/05/2020 | 7.0 |
| 13 | Convalescent plasma or hyperimmune immunoglobulin for people with COVID-19: a rapid review. | Cochrane Database of Systematic Reviews | 14/05/2020 | 11.1 |
| 14 | Clinical Outcomes in COVID-19 Patients Treated with Tocilizumab: An Individual Patient Data Systematic Review. | Journal of Medical Virology | 21/05/2020 | 4.7 |
| 15 | Convalescent plasma transfusion for the treatment of COVID-19: Systematic review. | Journal of Medical Virology | 01/05/2020 | 20.9 |
| 16 | Impact of corticosteroid therapy on outcomes of persons with SARS-CoV-2, SARS-CoV, or MERS-CoV infection: a systematic review and meta-analysis. | Leukemia | 05/05/2020 | 13.3 |
| 17 | Risks and Impact of Angiotensin-Converting Enzyme Inhibitors or Angiotensin-Receptor Blockers on SARS-CoV-2 Infection in Adults. | Annals of Internal Medicine | 15/05/2020 | 8.0 |
| 18 | Efficacy and safety of corticosteroids in COVID-19 based on evidence for COVID-19, other coronavirus infections, influenza, community-acquired pneumonia and acute respiratory distress syndrome: a systematic review and meta-analysis. | Canadian Medical Association Journal | 06/07/2020 | 10.2 |
| 19 | COVID-19 and treatment with NSAIDs and corticosteroids: Should we be limiting their use in the clinical setting?. | eCancer Medical Science | 30/03/2020 | 15.8 |
| 20 | A systematic review of the prophylactic role of chloroquine and hydroxychloroquine in coronavirus disease-19 (COVID-19). | International Journal of Rheumatic Disease | 13/04/2020 | 8.4 |
| 21 | Medical masks vs N95 respirators for preventing COVID-19 in healthcare workers: A systematic review and meta-analysis of randomized trials. | Influenza and other respiratory viruses | 21/04/2020 | 23.5 |
| 22 | An Updated Systematic Review of the Therapeutic Role of Hydroxychloroquine in Coronavirus Disease-19 (COVID-19). [Review] | Clinical Drug Investigation | 28/05/2020 | 5.7 |
| 23 | Non-steroidal anti-inflammatory drugs in management of COVID-19; a systematic review on current evidence. [Review] | The International Journal of Clinical Practice | 27/05/2020 | 1.9 |
| 24 | Hydroxychloroquine or Chloroquine for Treatment or Prophylaxis of COVID-19: A Living Systematic Review. | Annals of Internal Medicine | 27/05/2020 | 13.9 |
| 25 | Herbal Medicine for the Treatment of Coronavirus Disease 2019 (COVID-19): A Systematic Review and Meta-Analysis of Randomized Controlled Trials. [Review] | Journal of Clinical Medicine | 23/05/2020 | 3.4 |
| 26 | Antiviral therapy in management of COVID-19: a systematic review on current evidence. [Review] | Archives of Academic Emergency Medicine | 06/04/2020 | 3.2 |
| 27 | Efficacy and safety of antiviral treatment for COVID-19 from evidence in studies of SARSCoV-2 and other acute viral infections: a systematic review and meta-analysis. | Canadian Medical Association Journal | 06/07/2020 | 3.8 |
| 28 | The effect of corticosteroid treatment on patients with coronavirus infection: a systematic review and meta-analysis. | Journal of Infection | 10/04/2020 | 16.9 |
| 29 | Role of corticosteroid in the management of COVID-19: A systemic review and a Clinician's perspective. | Diabetes and Metabolic Syndrome | 27/06/2020 | 8.5 |
| 30 | High-flow nasal cannula for acute hypoxemic respiratory failure in patients with COVID-19: systematic reviews of effectiveness and its risks of aerosolization, dispersion, and infection transmission. | The Canadian Journal of Anesthesia | 15/06/2020 | 6.0 |
| 31 | Does Adding of Hydroxychloroquine to the Standard Care Provide any Benefit in Reducing the Mortality among COVID-19 Patients?: a Systematic Review. | Journal of Neuroimmune Pharmacology | 19/06/2020 | 2.4 |

*Citation rate calculated by dividing the total number of citations by the number of months since first publication
